# 85-Year-Old Postsurgical Complex Patient Successfully Managed Remotely at the Novel Mayo Clinic's Hospital at Home

**DOI:** 10.1155/2022/1439435

**Published:** 2022-02-25

**Authors:** Margaret R. Paulson, Ricardo A. Torres-Guzman, Francisco R. Avila, Karla Maita, John P. Garcia, Abdullah Eldaly, Luiza Palmieri-Serrano, Antonio J. Forte, Jonathan C. Thompson, Michael J. Maniaci

**Affiliations:** ^1^Division of Hospital Internal Medicine, Mayo Clinic Health Systems, Eau Claire, 2321 Stout Road, Menomonie, Wisconsin 54751, USA; ^2^Division of Plastic Surgery, Mayo Clinic, 4500 San Pablo Road, Jacksonville, Florida 32224, USA; ^3^Division of Orthopedics, Mayo Clinic Health Systems, 1400 Bellinger Stree, Eau Claire, Wisconsin 54703, USA; ^4^Division of Hospital Internal Medicine, Mayo Clinic, 4500 San Pablo Road, Jacksonville, Florida 32224, USA

## Abstract

An 85-year-old male presented to the podiatry clinic following a 1st to 5th left toe amputation as a complication of severe peripheral arterial disease and nonhealing wound despite endovascular intervention with an angiogram. At the visit, cellulitis with gangrene of the surgical site was noted. The patient was admitted to the brick and mortar (BAM) hospital and taken to surgery for a transmetatarsal amputation of the left limb. In the immediate postoperative period, the incisional margins appeared dusky creating concern for flap viability. The medical team recommended a vascular bypass versus a below-knee amputation. However, given the age, comorbidities, and nutritional status, the family refused further surgical intervention. As such, Mayo Clinic's home hospital program, Advanced Care at Home (ACH), was consulted for continued nonsurgical acute management at home. The patient was transferred to ACH and transported home three days after BAM admission to continue IV antibiotic therapy and wound care. Discharge from ACH occurred 11 days after admission to the BAM hospital. This case highlights the importance of developing health care alternatives to traditional hospitalization and demonstrates that ACH can manage highly complex, elder postoperative patients from the comfort of their homes.

## 1. Introduction

Increased hospital occupancy around the U.S. during the coronavirus pandemic meant that many surgical services required adaptions to traditional models of care for surgical patients requiring hospitalization. Elderly hospitalization is becoming more common in surgical departments. To date, almost two out of five surgical procedures are performed on patients over 65 years old [1]. Advanced Care at Home (ACH) is a unique hospital-at-home program for delivering acute and postacute care. This offering seeks to provide high-acuity care in the home setting while also aiming to reduce healthcare expenditures as well as decrease readmissions and healthcare-associated infections [2–7]. We report a case of a complex surgical patient managed at their home in northwest Wisconsin through the ACH centralized command center in Florida.

## 2. Case Presentation

An 85-year-old retired truck driver was seen by his family medicine physician after a recent hospitalization for atrial fibrillation with a rapid ventricular response. He had a past medical history of atrial fibrillation on anticoagulation, congestive heart failure, coronary artery disease status postcoronary artery bypass surgery, hypertension, aortic valve replacement, chronic kidney disease stage 3, obstructive sleep apnea, and peripheral vascular disease. Four months prior to his visit, he had undergone stenting of the right common iliac artery, angioplasty and stenting of the right superficial femoral artery, and angioplasty of the right tibioperoneal trunk and popliteal artery and ultimately required a right transmetatarsal amputation for osteomyelitis.

In the months leading up to his clinical visit, he started to develop digital gangrene in his left foot affecting the second and third digits. He had undergone an angiogram one month prior that showed severe stenosis of the left distal external and proximal common femoral arteries. Due to these findings, an atherectomy and drug-eluting balloon angioplasty were performed on the left distal iliac and common femoral arteries at that time. After that procedure, he was seen in follow-up in the podiatric clinic, where palpable pedal pulses were appreciated.

Now at his clinical visit his family medicine physician discovered worsening erythema in the left foot in the setting of persistent digital gangrene classified by the Society for Vascular Surgery Lower Extremity Threatened Limb: Wound, Ischemia, and foot Infection (SVS WIfI) as a W2I-fI1. Acute cellulitis of the left foot was diagnosed, and oral antibiotic management with cephalexin was initiated.

Two days after initiating management with cephalexin, the patient was assessed by his podiatrist who discovered full-thickness gangrene extending down to the base of the digit and onto the dorsal and plantar forefoot adjacent to the 2nd and 3rd digits (W3I-fI2). Due to the extension of the gangrene, a 1^st^ through 5^th^ toe amputation was deemed necessary and conducted at the brick and mortar (BAM) hospital at Mayo Clinic Health Systems at Eau Claire, Wisconsin, USA.

Four days postoperatively, the patient was seen in the podiatric clinic for routine follow-up. Upon physical exam, diffuse incisional dehiscence with progressive gangrenous changes was noted. The patient was admitted back to BAM hospital for IV antibiotics, vascular intervention, and definitive surgical management. Anesthesiology noted during its pre-operative risk assessment a Duke Activity Status index of 3.97 METs, a Gupta cardiac risk of myocardial infarction or cardiac arrest intraoperative or 30 days postoperative of 0.9%, a STOP-Bang Total Score of 5 (high risk for obstructive sleep apnea) and an ASA Physical Status Classification System of 4. During this hospitalization, the patient was also noted to both meet the ASPEN criteria of malnutrition as well as to have bacteremia with *Bacteroides fragilis*. Broad-spectrum antibiotics were first initiated and then subsequently downgraded to cefepime and metronidazole.

**Figure 1:**
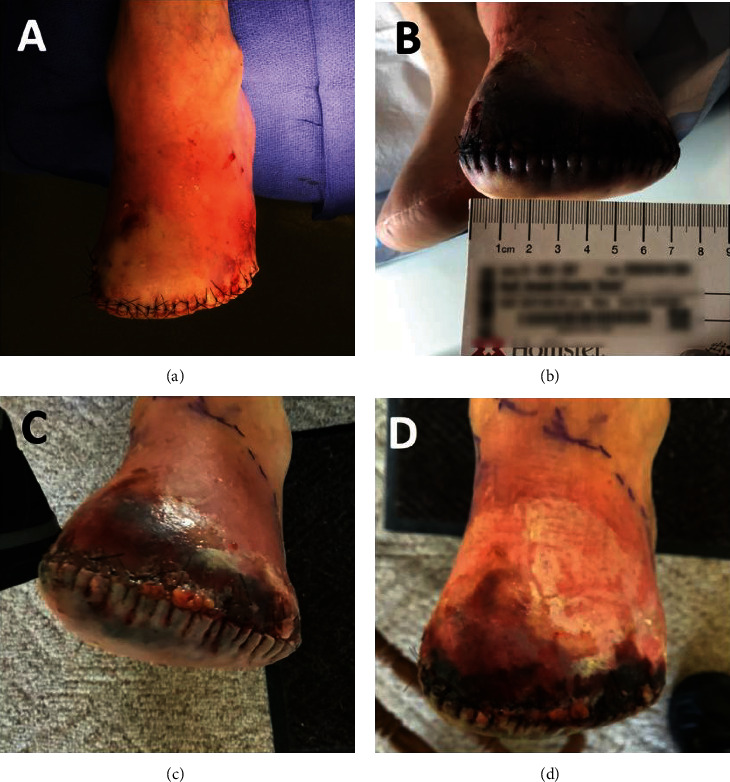
Follow-up pictures of the patient. (a) Intraoperative photograph of the transmetatarsal amputation. (b) ACH follow-up day three after transmetatarsal amputation. (c) The picture was taken on day 2 of the ACH program. (d) The image was taken on day 3 of the ACH program.

A transmetatarsal amputation was performed the day after readmission to BAM hospital. Marginal bleeding was appreciated intraoperatively with duskiness of the plantar flap and incisional margins, raising concern for insufficient vascular status to support healing of the amputation site (see Figure 1). Vascular Surgery was consulted for further discussion of revascularization options. An open femoral-popliteal vascular bypass was recommended versus the secondary option of a below-knee amputation. After considering the options presented in an informed consent discussion, the patient and family wished to pursue neither option and ultimately opted for palliative measures.

**Figure 2:**
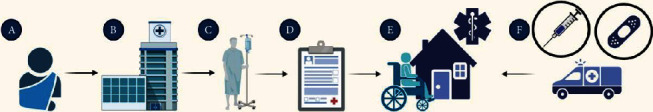
Patient transit flow chart. (a) The patient is sent to the ED after a clinic visit, where skin changes of the left foot were noticed. (b) The patient is hospitalized and evaluated by the podiatrist at Mayo Clinic Health Systems, Eau Claire, Wisconsin. (c) The patient is recommended to undergo the surgical procedure. (d) The family denies further procedures. The medical team consults the ACH team. The patient meets the criteria to be admitted to the program. (e) The patient is transported home, where the technological kit is given. (f) The command center located in Jacksonville, Florida, evaluated the patient daily. Additionally, a medical team visited the patient's home to administer antibiotics, perform a vascular assessment with a Doppler, and change the dressing of the wounds.

The medical team considered it appropriate to request a consultation by the ACH team to ensure that he was a potential candidate to continue acute management at home. The ACH team evaluated the patient at the bedside to determine eligibility based on insurance, demographic, social, and clinical criteria to be admitted into the program. The patient was transferred from the Medical Surgical floor to his home hospital three days after the transmetatarsal amputation. The technological kit consisting of a tablet device for video communication and data collection, a one way telephone to the centralized command center, and a personal emergency response system was given and tested. The patient and family received daily virtual rounds with the medical team coordinated by the single telemedicine command center located in Jacksonville, Florida, USA. Additionally, the patient had twice daily visits by a paramedic and nurse to evaluate peripheral pulses using a portable Doppler, change the dressings, take pictures for the command center, and administer the IV antibiotics (see Figure 2).

After the patient transferred to ACH, pain, erythema (W3I-fI2), and confusion increased progressively (see Figure 1). The patient, family, and healthcare team engaged in ongoing discussions about goals of care. Five days after transfer to the ACH program, the patient and the family decided to pursue hospice care. Seven days after admission, shared decision-making with the patient, family, and medical team, led to discharge from the ACH program and a smooth transition to hospice services.

## 3. Discussion

The traditional practice of medicine involves in-person consultation and examination. However, the current COVID-19 pandemic has obligated that many surgery services adapt quickly and implement telemedicine in their services by the degree of complexity of the patient and telehealth resources available at the different surgery service hospitals [8–10]. Telehealth has granted providers the ability to evaluate, diagnose, treat, and provide follow-up to surgical patients, particularly in places where resources and sub-specialists are insufficient. Moreover, telemedicine has been accepted by the public views as an acceptable substitute for in-person visits [9, 11], especially during the COVID-19, pandemic as shown in a recent publication by Sorensen et al. [9] where a 43-question survey was performed assessing respondents' attitudes toward telehealth for initial consultations with surgeons, both in the context of COVID-19 and during non-COVID-19 circumstances. The results showed that out of 1.827 responses, 86% of responders reported being satisfied (either extremely or somewhat) with telehealth encounters. The percentage of preference for virtual visits declined with the surgical procedure's complexity, even during the pandemic [9].

The ACH program was implemented at Mayo Clinic in July 2020. Patients in Florida and Wisconsin are admitted into the ACH program and monitored by a single telemedicine command center through telehealth. The command center conducts virtual rounds on the patient and delivers in-home care through a smart supply chain. The patient reported he had both daily virtual rounds by the Internal Medicine and Orthopedics hospital teams and in-home administered antibiotics, wound care, laboratory studies, and physical therapy. Furthermore, the ACH program differs from other hospital-at-home programs since it combines the two classifications of telemedicine in one. For instance, the two major classifications that characterize telemedicine are synchronous and asynchronous [12, 13]. Synchronous telemedicine or real-time telemedicine encompasses the use of telecommunication in real-time to simplify provider-patient interaction. On the other hand, asynchronous telemedicine uses data or images to transmit them electronically to be reviewed later. The ACH program allows providers and patients to interact using both types, synchronous and asynchronous, depending on the situation required.

A hospitalization is a threatening event for patients 65 years and older. The complexity of diseases and other health conditions make older patients prone to adverse hospital outcomes, including institutionalization, mortality, and functional decline [14, 15]. The mortality rate in these patients is approximately 20%; meanwhile, 30% of the survivors decrease their level of daily living activities (DLA) functioning three months after hospital discharge [16]. The ACH program seeks to provide high-acuity care in the home setting while also reducing healthcare expenditures and decreasing readmissions and healthcare-associated infections [2–7]. One of the most significant concerns when admitting the patient described in this case report to the ACH program was that given the patient's multiple comorbidities, his transition from the brick and mortar hospital to home would be hazardous. Nevertheless, not only the transition was successful, but 30-day readmission after hospital discharge was prevented [1].

Telemedicine in the surgical field exhibited the potential to increase productivity, follow-up rates, and access to healthcare by lessening travel-associated limitations [8, 17] and reducing costs [8, 18–20]. However, some of the limitations reported include the risk of misdiagnoses, information protection, technological literacy, and failure to identify abuse victims [8]. Additionally, the lack of in-person physical examination creates the need for a reliable virtual examination, especially in acute and complex conditions [8]. An online survey conducted in 2019 by Spear et al. [21] showed that out of 781 that experienced telemedicine reported the lack of hands-on care, intimacy, and technical difficulties as the main disadvantages [21]. There is a need to demonstrate that remote physical examination is reliable to follow-up complex postoperative patients as reported.

## 4. Conclusion

While hospital at home programs continue to gain popularity with both patients and providers, the limitations of successfully conducting telemedicine in postoperative patients, such as the equipment and supply chain organization required for both a complete daily remote evaluation as well as delivering high-acuity in-home care, still exist. However, the present case study highlights the ability of the Mayo Clinic's virtual hybrid hospital at home program to manage a complex postoperative patient with relative ease using telemedicine combined with coordinated in-person resources.

## Data Availability

Access to data is restricted to keep the patient's privacy. However, if deemed necessary, data will be provided by the corresponding author upon reasonable request after approval from the needed institutional committee.
